# Insulin resistance is associated with poor functional outcome after acute ischemic stroke in non-diabetic patients

**DOI:** 10.1038/s41598-020-80315-z

**Published:** 2021-01-13

**Authors:** Yoonkyung Chang, Chi Kyung Kim, Min-Kyung Kim, Woo‐Keun Seo, Kyungmi Oh

**Affiliations:** 1 Department of Neurology, Ewha Womans University Mokdong Hospital and Ewha University College of Medicine, 1071 Anyangcheon-ro, Yangcheon-gu, Seoul, South Korea; 2 Department of Neurology, Korea University Guro Hospital and Korea University College of Medicine, 148 Gurodong-Ro, Guro-gu, Seoul, 08308 South Korea; 3 Department of Neurology, Samsung Medical Center, Sungkyunkwan University School of Medicine, 81, Irwon‐ro, Gangnam‐gu, Seoul, South Korea

**Keywords:** Endocrinology, Neurology

## Abstract

Insulin resistance is associated with the occurrence of stroke and atherosclerotic disease. However, the relationship between insulin resistance and the prognosis of acute ischemic stroke in non-diabetic patients is unclear. We hypothesized that insulin resistance might affect short-term functional recovery after acute ischemic stroke in non-diabetic patients. Between May 2014 and December 2016, 1377 consecutive patients with acute ischemic stroke were enrolled from a prospectively maintained stroke registry. After excluding patients with transient ischemic attacks (TIA), pre-stroke disabilities, diabetes mellitus, and patients with incomplete evaluations, 517 patients were included in the study. The homeostasis model assessment of insulin resistance (HOMA-IR) score was used to evaluate the degree of insulin resistance. The patients with the highest quartile of log HOMA-IR index scores were younger and had higher fasting blood glucose, total cholesterol, triglycerides, low-density lipoprotein, and HbA1c levels. Multivariable logistic regression analysis revealed that log HOMA-IR scores were independently associated with poor prognosis after adjusting for age and sex and p < 0.1 in univariable analysis. Insulin resistance was associated with the poor functional outcome of non-diabetic stroke patients. This evidence supports treating insulin resistance in acute ischemic stroke patients with blood glucose levels within the normal range.

## Introduction

Insulin resistance is defined as the loss of insulin effects on target tissues, observed in patients with type 2 diabetes mellitus^1,2^. Insulin resistance results in impaired glucose utilization and increases hepatic glucose production. Among biomarkers of insulin resistance, the homeostasis model assessment-insulin resistance (HOMA-IR) index is an easy way to assess insulin resistance and is frequently used in epidemiologic studies^3^. The role of insulin in the central nervous system is an active research field. Studies have been published showing the association between insulin resistance and neurocognitive dysfunction^4,5^. In diabetic patients, the risk of dementia including Alzheimer's disease increases^6–9^. There are several roles of insulin and isoline resistance in the adult brain. Central nerves system insulin resistance causes diabetes and obesity through metabolic pathway homeostasis disruption^10,11^. There are also studies showing that insulin resistance affects the synaptic plasticity of the neuron^12^.

Meanwhile, it has long been known that hyperglycemia in stroke patients is associated with disease progression, neurologic deterioration and poor functional outcome^13,14^. Stroke treatment guidelines recommend adjusting blood sugar level to a normal range^15^, but treatment or assessment has not yet been recommended for insulin resistance. Recent studies have suggested that a strong relationship exists between insulin resistance and ischemic stroke. Insulin resistance has been reported to be associated with poor clinical outcomes after IV thrombolytic treatment^16,17^. Pioglitazone, a peroxisome proliferator-activated receptor-γ agonist formerly used in diabetic patients, improved insulin sensitivity and recurrent cardiovascular disease in patients with ischemic strokes or transient ischemic attacks^18,19^. Recently developed antidiabetic SGLT2 inhibitor, which affects insulin resistance^20,21^, was also confirmed to have a cardioprotective effect in diabetic patients^22^. A recent study in China reported that insulin resistance was associated with poor 1-year outcomes after acute ischemic strokes^23^. However, the reason for and mechanism of the association between insulin resistance and the prognosis of stroke patients has not been fully identified.

## Aim

This study hypothesized that insulin resistance might affect short-term functional recovery after acute ischemic strokes in non-diabetic patients. We aimed to determine whether this relationship was affected by stroke severity, stroke subtype, and clinical course.

## Methods

### Subjects

**Figure 1:**
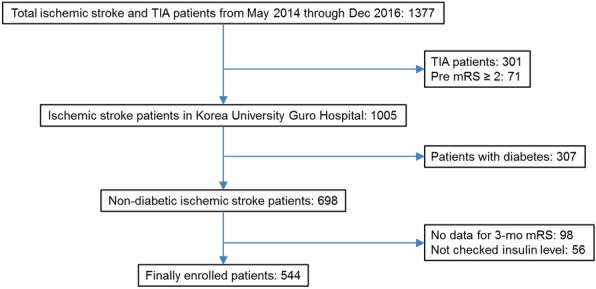
Flowchart of the enrolled patients in the study.

Between May 2014 and December 2016, 1377 patients with acute ischemic strokes within 7 days from symptom-onset were enrolled from a prospectively maintained stroke registry at our institution. In these 1377 patients, patients with transient ischemic attacks (TIA, n = 301) and pre-stroke disabilities (mRS score ≥ 2, n = 71) were excluded. After excluding the diabetic patients (n = 334) and those without 3 months of functional outcome data (n = 98) or insulin levels (n = 56), 517 patients were enrolled in the study (Fig. 1). This study was approved by the Institutional Review Board of our institution. Patient consent was waived due to the retrospective registry-based nature of the study.

### Insulin resistance

Fasting glucose and insulin levels were measured within 24 h of admission after an 8 h fast. Glucose level was measured using a Hitachi 747 chemistry analyzer (Boehringer Mannheim, Germany). The HOMA-IR was used to assess patients with insulin resistance. HOMA-IR scores were calculated by the following equation: [fasting insulin level (uU/mL)] × [fasting glucose level (mmol/L)]/22.5. HOMA-IR values were log-transformed due to their non-normal distribution. Subsequently, the log-transformed HOMA-IR values were categorized into quartiles (< 0.23, 0.23–0.36, 0.36–0.53, and > 0.53).

### Clinical and laboratory variables

Baseline demographics and previous history of hypertension, diabetes mellitus, dyslipidemia, coronary artery disease, peripheral artery disease, or cardiac arrhythmia were collected in all patients. Routine blood tests; chest X-rays; 12-lead electrocardiograms; transthoracic echocardiography; and brain imaging data, including computed tomography (CT), magnetic resonance imaging (MRI), and cerebral angiographic study using CT or/and MRI, were performed. Stroke severity was assessed by the National Institutes of Health Stroke Scale (NIHSS) scores, and the Trial of Org 10,172 in Acute Stroke Treatment (TOAST) classification was used to determine the subtypes of stroke etiology^24^. To assess the clinical course, the presence of END, defined as an increase in NIHSS score more than 2 points during admission, was collected. Functional outcome 3 months after the stroke onset was assessed through outpatient visits or centralized telephone follow-ups. Patient follow-ups were processed by a physician or a well-trained stroke nurse using a standardized interview protocol. Poor functional outcome was defined as a score of 3 or more on the mRS score.

### Statistical analysis

All statistical analyses were performed using SPSS (version 23.0, IBM Corp., Armonk NY, USA). The baseline characteristics were compared using Chi-squared (χ^2^) tests, independent t-tests, and analysis of variance (ANOVA) tests, as appropriate. The crude OR with 95% confidence intervals (CIs) for poor functional outcomes at 3 months were estimated by using the logistic regression model. The multivariable logistic regression model was used to further evaluate the relationship between HOMA-IR values and poor functional outcome. A two-tailed *p*-value of less than 0.05 was considered significant.

### Ethics approval and consent to participate

This study was approved by the Korea University of College of Medicine Institutional Review Board. Informed consent of study participants was waived because of the retrospective study design. All methods were performed by the relevant guidelines and regulations.

## Results

### Demographics and comparison of patients according to log HOMA-IR scores

**Figure 2:**
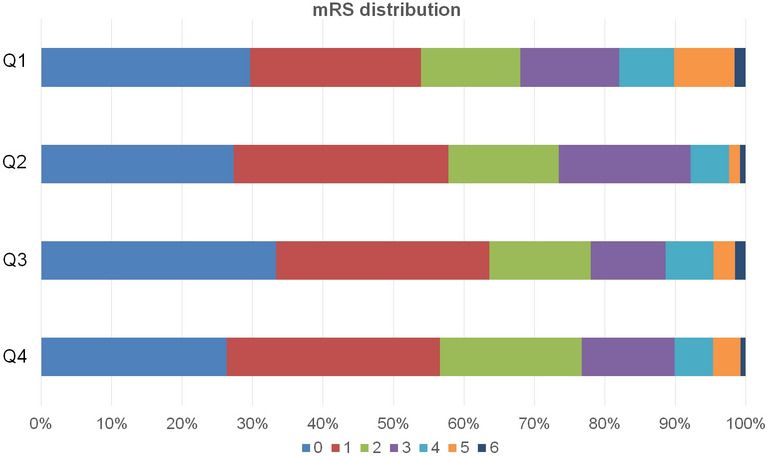
Modified Rankin Scale score distribution according to log homeostasis model assessment of insulin resistance score quartiles. Modified Rankin Scale score distribution shows higher score distributions in the highest quartile of log homeostasis model assessment of insulin resistance scores.

The mean age of the patients was 65.3 ± 13.5 years, and 65.2% were male. The median HOMA-IR value was 2.31 (interquartile range 1.68–3.42). When comparing patients according to the log HOMA-IR values divided into quartiles, age, fasting blood glucose levels, body mass index (BMI), lipid levels, and hemoglobin A1c values differed between quartiles. The highest quartile patients tended to be younger and had higher fasting blood glucose, total cholesterol, triglycerides, low-density lipoprotein, and hemoglobin A1c levels (Table 1). The presence of early neurologic deterioration (END) was not different between the groups. There was no statistical difference in the rate of poor prognosis among the log HOMA-IR quartiles. Still, the modified Rankin Scale (mRS) score distribution showed a tendency of higher mRS score distributions in the highest quartile (Fig. 2).

**Table 1:** Baseline characteristics according to log homeostasis model assessment of insulin resistance scores quartiles.

	HOMA-IR, quartiles				p-value
	Q1 (n = 129)	Q2 (n = 132)	Q3 (n = 128)	Q4 (n = 128)	
Age (years)	67.7 ± 14.2	65.9 ± 11.0	64.8 ± 13.2	62.6 ± 13.1	0.001
Male sex	79 (61.2)	91 (68.9)	80 (62.5)	87 (68.0)	0.467
Hypertension	58 (45.0)	70 (53.0)	73 (57.0)	72 (56.2)	0.194
Previous coronary artery disease	6 (4.7)	13 (9.8)	9 (7.0)	9 (7.0)	0.446
Previous stroke	11 (8.5)	15 (11.4)	13 (10.2)	9 (7.0)	0.648
Body mass index (kg/m^2^)	22.6 ± 2.9	24.0 ± 2.7	24.2 ± 4.1	25.2 ± 3.5	< 0.001
Fasting blood glucose (mg/dL)	93.1 ± 10.5	100.0 ± 13.9	107.2 ± 23.0	120.7 ± 26.1	< 0.001
Total cholesterol level (mg/dL)	184.2 ± 37.5	188.9 ± 42.0	191.5 ± 37.6	196.0 ± 40.4	0.015
Triglycerides level (mg/dL)	101.8 ± 54.1	123.4 ± 72.6	136.2 ± 87.8	154.0 ± 116.6	< 0.001
High-density lipoprotein cholesterol (mg/dL)	47.8 ± 12.9	46.2 ± 12.4	42.5 ± 10.0	44.9 ± 10.6	0.006
Low-density lipoprotein cholesterol (mg/dL)	108.6 ± 32.3	114.8 ± 37.0	117.1 ± 32.9	117.6 ± 33.6	0.029
Hemoglobin A1c (%)	5.5 ± 0.4	5.6 ± 0.4	5.6 ± 0.4	5.7 ± 0.4	0.001
Systolic blood pressure (mmHg)	150.5 ± 28.2	150.3 ± 28.6	150.5 ± 29.6	149.1 ± 28.4	0.723
Diastolic blood pressure (mmHg)	89.3 ± 13.6	88.0 ± 15.5	87.5 ± 16.0	89.7 ± 16.5	0.912
Initial NIHSS score, median (IQR)	3 (1–6)	2 (1–5)	3 (1–5)	3 (1–6)	0.146
**TOAST classification subtype**					0.953
Large artery atherosclerosis	30 (23.3)	32 (24.2)	37 (28.9)	43 (33.6)	
Cardioembolism	40 (31.0)	23 (17.4)	18 (14.1)	20 (15.6)	
Small vessel occlusion	25 (19.4)	39 (29.5)	37 (28.9)	25 (19.5)	
Undetermined	28 (21.7)	29 (22.0)	31 (24.2)	31 (24.2)	
Others	6 (4.7)	9 (6.8)	5 (3.9)	9 (7.0)	
Early neurological deterioration	4 (3.1)	12 (9.1)	6 (4.7)	8 (6.2)	0.197
Poor neurological outcome at 3 months	30 (23.3)	29 (22)	34 (26.6)	41 (32.0)	0.254

Data are expressed as the mean ± SD, or n (%).

HOMA-IR, homeostasis model assessment of insulin resistance scores; NIHSS, National Institutes of Health Stroke Scale (NIHSS); IQR, interquartile range; TOAST, the Trial of Org 10,172 in Acute Stroke Treatment.

### Association between log HOMA-IR values and short-term prognosis after ischemic stroke

Of all the subjects, 25.9% had poor prognoses. Table 2 shows a comparison of patients according to their prognosis 3 months after an acute ischemic stroke. The log HOMA-IR values were higher in patients with poor prognoses (*p* = 0.06). After adjustment for age, sex, and variables with *p*-values < 0.1 in univariate analysis, the log HOMA-IR scores were independently associated with poor prognosis (odds ratios (OR) = 3.877, 95% CI 1.461—10.288, *p* = 0.006) (Table 2). Subgroup analysis was performed on the severity of stroke (NIHSS score 0–8, ≥ 9), time of onset (within and after 24 h) and glucose level (≤ 100 and > 100), but no significant interactions were found (p for interaction 0.502, 0.229 and 0.524, respectively, Supplementary Tables 1–3).

**Table 2:** Factors associated with poor functional outcome at 3 months after ischemic stroke.

	Good prognosis (mRS < 3) (n = 383)	Poor prognosis (mRS ≥ 3) (n = 134)	p-value	Multivariable logistic regression	
				Model 1	Model 2
Age (years)	63.2 (12.9)	71.2 (11.5)	< 0.001	< 0.001	< 0.001
Male sex	251 (65.5)	86 (64.2)	0.858	0.036	0.031
Hypertension	192 (50.1)	81 (60.4)	0.050	0.661	0.597
Previous coronary artery disease	17 (4.4)	20 (14.9)	< 0.001	0.024	0.022
Previous stroke	28 (7.3)	20 (14.9)	0.015	0.326	0.312
HOMA-IR, median (IQR)	2.26 (1.66–3.23)	2.55 (1.75–3.99)	0.019		0.089
Log HOMA-IR, mean (SD)	0.38 ± 0.26	0.45 ± 0.26	0.006	0.006	
Body mass index (kg/m^2^)	24.0 ± 3.7	23.8 ± 2.9	0.527		
Fasting blood glucose (mg/dL)	104.4 ± 22.8	107.4 ± 19.0	0.144		
Total cholesterol level (mg/dL)	189.7 ± 38.5	191.4 ± 42.2	0.663		
Triglycerides level (mg/dL)	131.2 ± 89.6	121.8 ± 81.4	0.283		
High-density lipoprotein cholesterol (mg/dL)	45.6 ± 11.7	44.7 ± 11.6	0.449		
Low-density lipoprotein cholesterol (mg/dL)	113.0 ± 33.8	119.0 ± 34.6	0.080	0.001	< 0.001
Hemoglobin A1c (%)	5.6 ± 0.4	5.6 ± 0.4	0.413		
Systolic blood pressure (mmHg)	149.7 ± 29.8	151.4 ± 25.2	0.536		
Diastolic blood pressure (mmHg)	88.3 ± 15.9	89.4 ± 13.9	0.476		
Initial NIHSS score, median (IQR)	4 (2–5)	6 (3–13)	< 0.001	< 0.001	< 0.001
**TOAST classification subtype**			< 0.001	0.010	0.010
Large artery atherosclerosis	96 (25.1)	46 (34.3)			
Cardioembolism	71 (18.5)	30 (22.4)			
Small vessel occlusion	113 (29.5)	13 (9.7)			
Undetermined	81 (21.1)	38 (28.4)			
Others	22 (5.7)	7 (5.2)			
Early neurological deterioration	11 (2.9)	19 (14.2)	< 0.001	< 0.001	< 0.001

Data are expressed as the mean ± SD, or n (%).

mRS, modified Rankin Scale; HOMA-IR, homeostasis model assessment of insulin resistance scores; IQR, interquartile range; NIHSS, National Institutes of Health Stroke Scale (NIHSS); TOAST, the Trial of Org 10,172 in Acute Stroke Treatment.

## Discussion

Our study showed that log HOMA-IR scores were significantly associated with non-diabetic ischemic stroke patients' poor functional outcome. This association remained statistically significant even the age of the highest quartiles of log HOMA-IR was youngest among quartiles, and the relationship was sustained after adjusting for cardiovascular risk factors and lipid profile abnormalities.

The most significant factors influencing stroke prognosis are the severity of the initial stroke severity and worsening of neurologic symptoms. But in this study, the initial stroke severity and the presence of END were not different between the HOMA-IR quartiles. Additionally, no interaction was observed in the subgroup analysis for stroke severity and log HOMA IR. These results indicate that the detrimental effects of insulin resistance affect the recovery phase of acute ischemic stroke and are not associated with exacerbating pre-existing disability. Since insulin resistance has been known to affect neuroplasticity in patients with diabetes^12^, our study result is in line with previous studies. A recent registry-based cohort study in Japan also reported that HOMA-IR scores were related to the poor functional outcome at 1 year^25^. They showed no associations between insulin resistance and recurrent stroke or mortality. Since we hypothesized that insulin resistance affects recovery after stroke, these results align with our study. Meanwhile, another study in China reported that the HOMA-IR index scores were associated with increased mortality, recurrent stroke, and short-term poor outcomes^23^. The differences between the study results may be due to different study populations.

Some hypotheses may explain the association between insulin resistance and poor patient outcome after an ischemic stroke. One of them involves the concept of synaptic plasticity. Synaptic plasticity is a neuron's ability to change the synapse in response to external stimuli and activity. In the brain, the insulin/IGF receptor signaling pathway maintains the balance between neuroprotective and neurotoxic effects^26–28^. Insulin resistance is defined as a loss of this function in insulin ligands. Subsequently, when the balance is broken, it causes changes in the neurons' survival and synaptic plasticity. Likewise, the brain's synaptic plasticity decreases in stroke patients with high insulin resistance, which interferes with its recovery from the primary insult. Second, insulin resistance in muscles may have contributed to the poor prognosis of these patients. Type 2 diabetes mellitus patients evolve whole-body insulin resistance, and insulin resistance in skeletal muscles reduces glucose transport pathways, which results in excessive reactive oxygen species and mitochondrial dysfunction^29,30^. This may interrupt recovery after an acute ischemic stroke. Third, endothelial damage might play a role. Endothelial function is related to vascular reactivity in the cerebral circulation^31^. Insulin and insulin resistance affect vascular endothelium^31–33^. Furthermore, insulin resistance is a risk factor for atherosclerosis^34,35^. Endothelial dysfunctions, decreased vascular reactivity, and enhanced atherosclerosis might cause recurrent stroke and delayed restoration of function.

Our study had some strengths. First, we demonstrated that the worsening effect of insulin resistance on ischemic stroke impacted the recovery phase. This finding indicates that we should treat insulin resistance itself, apart from diabetes mellitus, especially in the subacute stage of an acute ischemic stroke. Second, unlike previous studies, we thoroughly investigated the risk factors, including laboratory and clinical aspects, associated with poor prognoses. Body mass index, individual lipid levels, and blood pressure levels were collected and adjusted for in the multivariate analysis, and the association between insulin resistance and the poor clinical outcome remained strong. There were several limitations to this study. First, the HOMA-IR scores were only determined once, within 24 h of admission. This might not reflect the exact status of insulin resistance during the recovery period. Second, the HOMA-IR index scores for insulin resistance mainly reflect resistance in hepatic metabolism. Third, this was a single-center data review from a comprehensive stroke center in Korea. Consequently, the results cannot be generalized to other populations and races.

## Conclusion

Insulin resistance measured by the HOMA-IR index was associated with the poor functional outcome of non-diabetic stroke patients. This finding may strengthen the need to treat insulin resistance itself in acute ischemic stroke patients with blood glucose levels within the normal range.

## Supplementary Information

Supplementary Information.

## References

[1] Kahn SE (2001). Clinical review 135: The importance of beta-cell failure in the development and progression of type 2 diabetes. J. Clin. Endocrinol. Metab..

[2] Bergman RN, Ader M (2000). Free fatty acids and pathogenesis of type 2 diabetes mellitus. Trends Endocrinol. Metab..

[3] Matthews DR (1985). Homeostasis model assessment: Insulin resistance and beta-cell function from fasting plasma glucose and insulin concentrations in man. Diabetologia.

[4] Gao C, Liu Y, Li L, Hölscher C (2013). New animal models of Alzheimer's disease that display insulin desensitization in the brain. Rev. Neurosci..

[5] Stoeckel LE (2016). Complex mechanisms linking neurocognitive dysfunction to insulin resistance and other metabolic dysfunction. F1000Research.

[6] de la Monte SM (2009). Insulin resistance and Alzheimer's disease. BMB Rep..

[7] Teixeira MM (2020). Association between diabetes and cognitive function at baseline in the Brazilian Longitudinal Study of Adult Health (ELSA-Brasil). Sci. Rep..

[8] Ott A (1999). Diabetes mellitus and the risk of dementia: The Rotterdam Study. Neurology.

[9] Biessels GJ, Staekenborg S, Brunner E, Brayne C, Scheltens P (2006). Risk of dementia in diabetes mellitus: A systematic review. Lancet. Neurol..

[10] Brüning JC (2000). Role of brain insulin receptor in control of body weight and reproduction. Science.

[11] Lin X (2004). Dysregulation of insulin receptor substrate 2 in beta cells and brain causes obesity and diabetes. J. Clin. Investig..

[12] Grillo CA (2015). Hippocampal insulin resistance impairs spatial learning and synaptic plasticity. Diabetes.

[13] Lindsberg PJ, Roine RO (2004). Hyperglycemia in acute stroke. Stroke.

[14] Savopoulos C (2017). Is management of hyperglycaemia in acute phase stroke still a dilemma?. J. Endocrinol. Investig..

[15] Powers WJ (2019). Guidelines for the early management of patients with acute ischemic stroke: 2019 update to the 2018 guidelines for the early management of acute ischemic stroke: A guideline for healthcare professionals from the American Heart Association/American Stroke Association. Stroke.

[16] Bas DF, Ozdemir AO, Colak E, Kebapci N (2016). Higher insulin resistance level is associated with worse clinical response in acute ischemic stroke patients treated with intravenous thrombolysis. Transl. Stroke Res..

[17] Calleja AI (2011). Insulin resistance is associated with a poor response to intravenous thrombolysis in acute ischemic stroke. Diabetes Care.

[18] Kernan WN (2003). Pioglitazone improves insulin sensitivity among nondiabetic patients with a recent transient ischemic attack or ischemic stroke. Stroke.

[19] Kernan WN (2016). Pioglitazone after ischemic stroke or transient ischemic attack. N. Engl. J. Med..

[20] Sa-Nguanmoo P (2017). SGLT2-inhibitor and DPP-4 inhibitor improve brain function via attenuating mitochondrial dysfunction, insulin resistance, inflammation, and apoptosis in HFD-induced obese rats. Toxicol. Appl. Pharmacol..

[21] Han S (2008). Dapagliflozin, a selective SGLT2 inhibitor, improves glucose homeostasis in normal and diabetic rats. Diabetes.

[22] Zelniker TA (2019). SGLT2 inhibitors for primary and secondary prevention of cardiovascular and renal outcomes in type 2 diabetes: A systematic review and meta-analysis of cardiovascular outcome trials. Lancet.

[23] Jing J (2017). Insulin resistance and prognosis of nondiabetic patients with ischemic stroke: The ACROSS-China Study (abnormal glucose regulation in patients with acute stroke across China). Stroke.

[24] Adams HP (1993). Classification of subtype of acute ischemic stroke. Definitions for use in a multicenter clinical trial. TOAST. Trial of Org 10172 in acute stroke treatment. Stroke.

[25] Ago T (2018). Insulin resistance and clinical outcomes after acute ischemic stroke. Neurology.

[26] Yamaguchi F (1990). Insulin-like growth factor I (IGF-I) distribution in the tissue and extracellular compartment in different regions of rat brain. Brain Res..

[27] Baskin DG, Figlewicz DP, Woods SC, Porte D, Dorsa DM (1987). Insulin in the brain. Annu. Rev. Physiol..

[28] Le Roith D (1983). Insulin in brain and other extrapancreatic tissues of vertebrates and nonvertebrates. Adv. Metab. Disord..

[29] Zierath JR, Krook A, Wallberg-Henriksson H (2000). Insulin action and insulin resistance in human skeletal muscle. Diabetologia.

[30] Di Meo S, Iossa S, Venditti P (2017). Skeletal muscle insulin resistance: Role of mitochondria and other ROS sources. J. Endocrinol..

[31] Prakash K, Chandran DS, Khadgawat R, Jaryal AK, Deepak KK (2016). Correlations between endothelial function in the systemic and cerebral circulation and insulin resistance in type 2 diabetes mellitus. Diabetes Vasc. Dis. Res..

[32] Muniyappa R, Quon MJ (2007). Insulin action and insulin resistance in vascular endothelium. Curr. Opin. Clin. Nutr. Metab. Care.

[33] Hsueh WA, Law RE (1999). Insulin signaling in the arterial wall. Am. J. Cardiol..

[34] Sourij H (2008). Insulin resistance as a risk factor for carotid atherosclerosis: A comparison of the Homeostasis Model Assessment and the short insulin tolerance test. Stroke.

[35] Gage MC (2013). Endothelium-specific insulin resistance leads to accelerated atherosclerosis in areas with disturbed flow patterns: A role for reactive oxygen species. Atherosclerosis.

