# Supplementary material for: Insulin resistance is associated with poor functional outcome after acute ischemic stroke in non-diabetic patients
Source: Sci Rep. 2021 Jan 13;11:1229. doi: 10.1038/s41598-020-80315-z (PMC7806587; doi:10.1038/s41598-020-80315-z)
Supplement: Supplementary file 1 — Supplementary Information. [file 41598_2020_80315_MOESM1_ESM.docx]

**Title:** Insulin resistance is associated with poor functional outcome after acute ischemic stroke in non-diabetic patients**.**

**Authors:**

Yoonkyung Chang, MD^1*^, Chi Kyung Kim, MD, PhD^2*^, Min-Kyung Kim, MD^2^, Woo‐Keun Seo, MD, PhD^3^, Kyungmi Oh, MD, PhD^2^

^1^Department of Neurology, Ewha Womans University Mokdong Hospital and Ewha University College of Medicine, 1071 Anyangcheon-ro, Yangcheon-gu, Seoul, South Korea

^2^Department of Neurology, Korea University Guro Hospital and Korea University College of Medicine,148 Gurodong-ro, Guro-gu, Seoul, South Korea

^3^Department of Neurology, Samsung Medical Center, Sungkyunkwan University School of Medicine, 81, Irwon‐ro, Gangnam‐gu, Seoul, South Korea

^*^ these authors contributed equally to this work

**Correspondence to:**

Kyungmi Oh, MD, PhD

Department of Neurology, Korea University Guro Hospital and Korea University College of

Medicine, 148 Gurodong-ro, Guro-gu, Seoul 08308, South Korea

Tel: +82-2-2626-3170/Fax: +82-2-2626-2249/E-mail: [okyungmi@korea.ac.kr](mailto:okyungmi@korea.ac.kr)

Supplementary Table 1. Multivariate analysis of log HOMA-IR poor functional outcome regarding onset time to admission.

|  | Poor functional outcome | | |
| --- | --- | --- | --- |
|  | OR (95% CI) | *p* | *p* for interaction |
| log HOMA-IR |  |  | 0.229 |
| admission within 24 hours | 5.007 (1.581-15.856) | 0.006 |  |
| after 24 hours | 1.892 (0.283-12.637) | 0.511 |  |

OR, odds ratio; CI, confidence interval; HOMA-IR, homeostasis model assessment of insulin resistance scores

Supplementary Table 2. Multivariate analysis of log HOMA-IR poor functional outcome regarding stroke severity.

|  | Poor functional outcome | | |
| --- | --- | --- | --- |
|  | OR (95% CI) | *p* | *p* for interaction |
| log HOMA-IR |  |  | 0.502 |
| NIHSS 0-8 | 3.758 (1.256-11.246) | 0.018 |  |
| HIHSS ≥9 | 10.123 (1.178-87.002) | 0.035 |  |

OR, odds ratio; CI, confidence interval; HOMA-IR, homeostasis model assessment of insulin resistance scores

Supplementary Table 3. Multivariate analysis of log HOMA-IR poor functional outcome regarding glucose level.

|  | Poor functional outcome | | |
| --- | --- | --- | --- |
|  | OR (95% CI) | *p* | *p* for interaction |
| log HOMA-IR |  |  | 0.524 |
| Glucose level ≤100 | 4.740 (0.721-31.156) | 0.105 |  |
| Glucose level >100 | 3.500 (0.971-12.624) | 0.056 |  |

OR, odds ratio; CI, confidence interval; HOMA-IR, homeostasis model assessment of insulin resistance scores
